# Expression and Immunogenicity of M2e Peptide of Avian Influenza Virus H5N1 Fused to Ricin Toxin B Chain Produced in Duckweed Plants

**DOI:** 10.3389/fchem.2018.00022

**Published:** 2018-02-13

**Authors:** Aleksey Firsov, Irina Tarasenko, Tatiana Mitiouchkina, Lyubov Shaloiko, Oleg Kozlov, Leonid Vinokurov, Ekaterina Rasskazova, Arkadii Murashev, Alexander Vainstein, Sergey Dolgov

**Affiliations:** ^1^Institute of Bioorganic Chemistry (RAS), Moscow, Russia; ^2^Robert H. Smith Faculty of Agriculture, Food and Environment, Hebrew University of Jerusalem, Rehovot, Israel

**Keywords:** duckweed, avian influenza, peptide M2e, ricin B subunit, mice immunization, edible vaccine

## Abstract

The amino acid sequence of the extracellular domain of the virus-encoded M2 matrix protein (peptide M2e) is conserved among all subtypes of influenza A strains, enabling the development of a broad-range vaccine against them. We expressed M2e from avian influenza virus A/chicken/Kurgan/5/2005 (H5N1) in nuclear-transformed duckweed plants for further development of an avian influenza vaccine. The 30-amino acid N-terminal fragment of M2, including M2e (denoted M130), was selected for expression. The M2e DNA sequence fused in-frame to the 3′ end of ricin toxin B chain (RTB) was cloned under control of the CaMV 35S promoter into pBI121. The resulting plasmid was used for duckweed transformation, and 23 independent transgenic duckweed lines were obtained. Asialofetuin-binding ELISA of protein samples from the transgenic plants using polyclonal anti-RTB antibodies confirmed the expression of the RTB–M130 fusion protein in 20 lines. Quantitative ELISA of crude protein extracts from these lines showed RTB–M130 accumulation ranging from 0.25–2.5 μg/g fresh weight (0.0006–0.01% of total soluble protein). Affinity chromatography with immobilized asialofetuin and western blot analysis of protein samples from the transgenic plants showed expression of fusion protein RTB–M130 in the aggregate form with a molecular mass of about 70 kDa. Mice were immunized orally with a preparation of total soluble protein from transgenic plants, receiving four doses of 7 μg duckweed-derived RTB–M130 each, with no additional adjuvant. Specific IgG against M2e was detected in immunized mice, and the endpoint titer of nti-M2e IgG was 1,024. It was confirmed that oral immunization with RTB-M130 induces production of specific antibodies against peptide M2e, one of the most conserved antigens of the influenza virus. These results may provide further information for the development of a duckweed-based expression system to produce a broad-range edible vaccine against avian influenza.

## Introduction

Avian influenza is one of the most important diseases in poultry (Swayne et al., 2013). Mass vaccination of domestic and wild birds is the most effective method of preventing this disease (Porter, 2015), hence the importance of inexpensive and easy-to-use vaccines. The ideal vaccine should be able to induce cross-protection against various disease-causing avian influenza strains; it should also be deliverable via mass-immunization methods, such as feeding or in the drinking water; finally, it should be inexpensive to manufacture and convenient for transportation and storage. Plant-produced edible vaccines might be able to meet all of these requirements (Shahid and Daniell, 2016).

Conventional influenza vaccines are based on immunity to the surface viral proteins hemagglutinin and neuraminidase. However, due to antigenic drift, the antigenic properties of these proteins are constantly changing, calling for vaccine updating on a regular basis (Porter, 2015). In this regard, considerable effort is now being focused on the development of vaccines based on conserved viral antigens—the so-called “universal” vaccine—that are effective against many circulating strains. The extracellular domain of matrix protein 2 (M2), termed peptide M2e, and the hemagglutinin stalk region have attracted general interest as antigens for “universal” vaccine design (Oxford, 2013; Wong and Webby, 2013; Hefferon, 2014; Scorza et al., 2016).

Influenza M2 is a transmembrane protein consisting of 97 amino acids; the 23 amino acids at the N terminus form the M2e ectodomain (Pinto and Lamb, 2006). M2 functions as a homotetrameric ion channel and plays an important role in uncoating the virus after its entry. Blocking the ion channel prevents infection of a host cell with the virus (Knipe et al., 2001). In influenza A viruses, the 9 N-terminal amino acids of M2e are completely conserved, while there are only minor changes in the membrane-proximal region. Due to its high conservation among influenza A viruses, M2e has been considered a promising target for inducing cross-protection against different influenza strains (Ito et al., 1991; Fiers et al., 2004; Tumpey et al., 2005). The possibility of developing M2e-based influenza vaccines has been repeatedly confirmed. Some of these vaccines are already in various stages of clinical trials (Oxford, 2013; Zhang et al., 2014; Zheng et al., 2014). Unfortunately, the native form of M2e is poorly immunogenic (Ebrahimi and Tebianian, 2011). One of the strategies for increasing its immunogenicity is to fuse it with adjuvant molecules. The most commonly used adjuvants are hepatitis B core antigen and heat-shock protein 70 of *Mycobacterium tuberculosis* (Oxford, 2013). Other adjuvants, such as *Escherichia coli* flagellin, CD154 from *Salmonella enteritidis* strains and CpG oligonucleotide have also been successfully used to enhance the immunogenicity of the M2e peptide (Ebrahimi and Tebianian, 2011; Oxford, 2013).

Among the promising adjuvants for the development of edible vaccines are plant lectins (Granell et al., 2011; Souza et al., 2013). Plant lectins are a class of carbohydrate-binding proteins; they bind specifically and reversibly to carbohydrate moieties on cell surfaces without biochemical modifications. This ability to bind cell-surface carbohydrate moieties, particularly the M cells on Peyer's patches of mucosal surfaces, has prompted interest in lectins as antigen-delivery agents for mucosal vaccination (Granell et al., 2010). The few experiments involving the immunization of animals with different antigens fused to the nontoxic subunit B of the plant lectin ricin (RTB) have proven the efficacy of RTB as a strong mucosal adjuvant, as low doses of it enhance the immune response to fusion proteins (Choi et al., 2006; Donayre-Torres et al., 2009; Singh et al., 2015). The effectiveness of RTB as an adjuvant is equal to that of subunit B of cholera toxin (Medina-Bolivar et al., 2003). The antigens used in Choi et al. (2006) and Donayre-Torres et al. (2009) were produced in *E. coli*. Medina-Bolivar et al. (2003) and Singh et al. (2015) used antigens expressed in hairy root cultures—tobacco and tomato, respectively. Choi et al.'s (2006) study is the only one in which the antigen was administered orally.

There have been a number of reports on M2e peptide expression in plants (Nemchinov and Natilla, 2007; Denis et al., 2008; Meshcheryakova et al., 2009; Tyulkina et al., 2011; Kang et al., 2012; Petukhova et al., 2014; Mbewana et al., 2015; Tsybalova et al., 2015; Mardanova et al., 2016). In all of them, the M2e was expressed in transient virus vector-based systems. Plant-produced M2e peptide as part of a virus-like particle or fusion protein provided partial (Meshcheryakova et al., 2009; Mardanova et al., 2016) or complete (Denis et al., 2008; Mbewana et al., 2015; Tsybalova et al., 2015) protection of mice against influenza virus challenge. In two of these studies, the M2e peptide was fused to adjuvant proteins—hepatitis B virus core antigen (Tsybalova et al., 2015) or *Salmonella typhimurium* flagellin (Mardanova et al., 2016). In the other experiments, the animals were immunized with virus-like particles that included the M2e peptide but without fusion to the adjuvant protein. The animals in those experiments were immunized parenterally. Since the production of and trials with stably transformed plants are lengthy processes during which vaccines based on hemagglutinin and neuraminidase may lose their relevance, most of these studies were based on transiently expressed antigen. The M2e sequence has remained almost unchanged since 1918 (Tumpey et al., 2005), making it highly suitable for the development of edible vaccines based on nuclear-transformed plants that do not require the expensive industrial-scale facilities necessary for transient expression systems.

Duckweed (*Lemna minor*) is a highly attractive plant system for stable expression of biotechnological products (Gasdaska et al., 2003; Everett et al., 2012). Rapid growth (36 h doubling time) in liquid media, high protein content (up to 45% dry weight), and effective and inexpensive cultivation in bioreactors of various types make this aquatic monocotyledonous plant highly useful for the production of commercially important products in a well-controlled format. The ability to cultivate duckweed in contained systems enables overcoming an important limitation to the commercialization of plant expression systems—potential accidental release of genetically modified plants into the environment during the cultivation of transgenic plants in the field or greenhouse. Several commercially important proteins have already been successfully expressed in duckweed plants: the endoglucanase E1 from *Acidothermus cellulolyticus* (Sun et al., 2007), and monoclonal antibodies against CD20, CD30, and interferon α2b (Cox et al., 2006). Protective antigen of swine epidemic diarrhea (Ko et al., 2011), tuberculosis antigens ESAT6 and Ag85B (Peterson et al., 2015), and hemagglutinin of avian influenza virus H5N1 (Bertran et al., 2015; Huong et al., 2015) have also been successfully expressed in these plants.

Thus, although the potential of plant-derived RTB as an adjuvant has already been confirmed, the possibility of its oral delivery has not yet been studied. Moreover, the plant-produced M2e was not used for oral immunization in any of the above studies. The aims of this work were to explore the feasibility of expressing M2e fused to RTB (RTB–M130) in nuclear-transformed duckweed plants and to evaluate the immunogenicity of this fusion protein. We report the successful *Agrobacterium*-mediated transformation of duckweed and the expression of peptide M2e as part of the fusion protein RTB–M130 in transgenic plants. We also show that a preparation of partially purified total protein from transgenic plants was immunogenic when ingested, activating an immune response in mice.

## Materials and methods

### *Agrobacterium*-mediated transformation of duckweed and vector construction

*In-vitro* culture of duckweed (*L. minor* L.) from Oka River was used in the experiments. *Agrobacterium*-mediated duckweed callus transformation, selection and regeneration of the transformants, and their cultivation *in vitro* were carried out as described previously (Firsov et al., 2015).

A fragment comprising 30 N-terminal amino acid residues of the M2 protein of avian influenza virus A/chicken/Kurgan/5/2005 (H5N1) (GenBank accession no. DQ449633.1), including 23 amino acids of peptide M2e (termed M130), was selected for expression in transgenic duckweed. The DNA sequence of the M130 peptide was optimized for expression in duckweed by codon usage in *Lemna gibba* (http://www.kazusa.or.jp/codon/).

**Figure 1:**
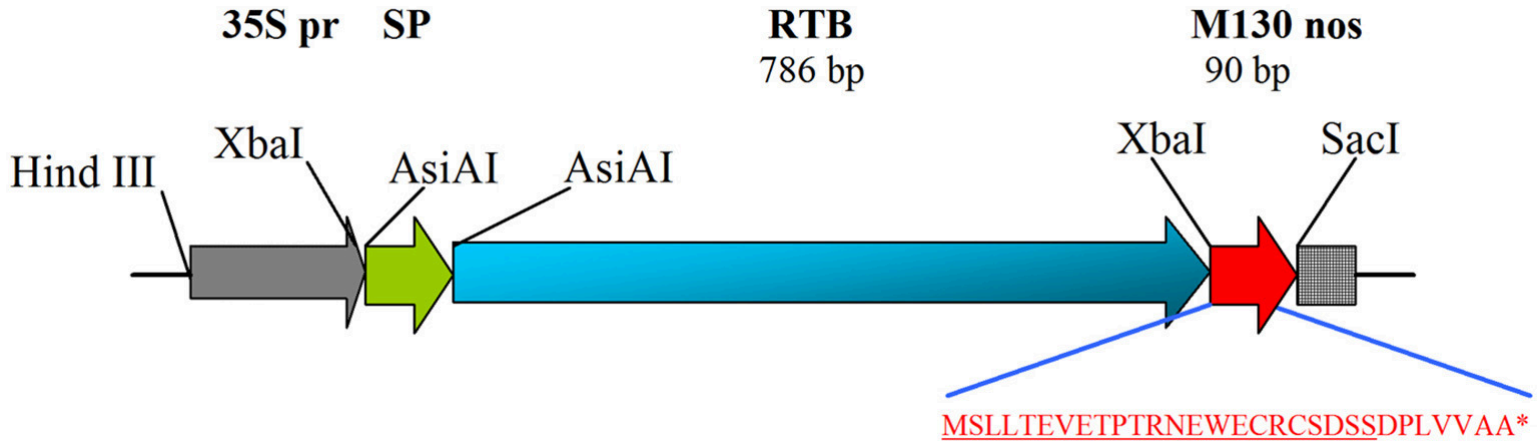
Schematic depiction of the expression cassette of plasmid pRTB–M130. The amino acid sequence of M130 peptide is shown. 35S pr, CaMV 35S promoter; SP, N-terminal signal peptide of tobacco PR1a protein; RTB, subunit B of ricin; M130, N-terminal fragment of the M2 protein of avian influenza virus containing peptide M2e (underlined); nos, nopaline synthase terminator. The sizes of RTB and M130 are indicated. ^*^Denotes a stop codon, i.e., end of the amino acid sequence of RTB-M130 protein.

PCR amplification of the RTB-encoding nucleotide sequence (GenBank accession no. X03179) was performed using primers RTB/F and RTB/R (the PCR regimes, primer sequences and cloning sites are presented in Table S1). Total DNA isolated from castor bean leaves was used as the template. The amplified DNA fragment was cloned into the intermediate vector pUC18 and sequenced. The nucleotide sequence of the N-terminal signal peptide of tobacco protein PR1a (GenBank accession no. X12737) was amplified from genomic tobacco DNA using primers RBspF and RBspR. The nucleotide sequence of RTB was cloned in fusion with the 5′ end of M130 and 3′ end of the signal peptide of PR1a into vector pBI121, replacing the β-glucuronidase gene (Figure 1). After sequencing, the resulting plasmid (pRTB-M130) was transferred into *Agrobacterium tumefaciens* CBE21 and used for transformation.

In the immunization experiments, we also used transgenic duckweed plants expressing the fusion protein M2e–β-glucuronidase (M2e–GUS; Firsov et al., 2015). Transgenic line G54 with the highest accumulation of M2e–GUS (0.97 mg/g duckweed fresh weight [FW], corresponding to 1.96% of total soluble protein [TSP]) was used. All transgenic lines were grown in 250-ml flasks containing 50 ml of liquid hormone-free MS medium without antibiotics (LHFM medium). Approximately 0.3 g duckweed was inoculated into each flask. The plants were subcultured in fresh medium every 3 weeks. The cultivation of duckweed lines was carried out at 25 ± 1°C, under a 16/8 h photoperiod and illumination intensity of 1.5 W/m^2^.

### PCR and southern blot analyses

For PCR and Southern blot analyses, the genomic DNA of duckweed was isolated from kanamycin-resistant and non-transformed control plants using the method of Dellaporta et al. (1983). PCR analysis of putatively transgenic plants was performed using primers 5727 and M130r (Table S1). For the Southern blot, duckweed genomic DNA (50 μg) was digested overnight at 37°C with 100 U HindIII which cut the T-DNA of pRTB–M130 at a single position (5′ end of the CaMV 35S promoter). After gel electrophoresis, the digestion products were transferred and immobilized onto Hybond+ membrane (Amersham, USA). The DNA probe was constructed by PCR using plasmid pRTB–M130 as the template and primers RBspF and M130r, amplifying the nucleotide sequence of fusion protein RTB–M130. Probe DNA (1,003 bp) was labeled with alkaline phosphatase using the AlkPhos Direct Labeling Kit (Amersham Bioscience, USA). Prehybridization, hybridization (overnight at 60°C) with alkaline phosphatase-labeled probe, and subsequent washings of the membrane were carried out according to the AlkPhos Direct Labeling Kit protocol. Detection was performed using CDP-Star detection reagent following the manufacturer's directions (Amersham Bioscience).

### Asialofetuin-binding ELISA

RTB–M130 recombinant protein was quantified in protein extracts of transgenic duckweed plants by asialofetuin-binding assay (Dawson et al., 1999). TSP was extracted immediately prior to the analysis. Duckweed plants (1.0 g) were ground in liquid nitrogen. The ground material was resuspended in an equal volume of phosphate buffer saline (PBS, pH 7.4) containing 0.05% (v/v) Tween 20 (PBST buffer). Total proteins were extracted for 20 min at 4°C, then centrifuged for 10 min at 16,000 g, 4°C and the supernatant was taken for further analysis. Protein concentration was measured by DC protein assay (BioRad, USA).

Analyzed protein samples (100 μl/well) were added to the wells with immobilized asialofetuin. Rabbit anti-RTB polyclonal antibody (AbCam, UK) was used as the primary antibody, and anti-rabbit IgG conjugated to alkaline phosphatase (Pierce, USA) as the secondary antibody. Antibodies were diluted in blocking buffer, 1:1,000 for primary and 1:2,000 for secondary antibodies. Phosphatase Substrate Kit (Pierce) was used, and the reaction was performed as per the manufacturer's instructions. The reaction was stopped after 20 min at 25°C and absorbance was measured at 405 nm.

Ricin, extracted from castor seeds following Waller and Negi's (1958), was used as a standard for the quantification of RTB–M130 accumulation in transgenic plants. The amount of plant-expressed RTB–M130 was estimated based on reference ricin standards taking into account differences in their molecular mass (32.6 kDa for RTB–M130 and 64.2 kDa for ricin).

### Affinity chromatography with immobilized asialofetuin

Affinity chromatography with immobilized asialofetuin was carried out by the method described in Donayre-Torres et al. (2009). In this experiment, we used transgenic line 81 and non-transformed duckweed as a negative control. The duckweed plants (30 g FW) were ground in liquid nitrogen. The ground material was resuspended in three volumes of extraction buffer containing 50 mM Tris–HCl, pH 7.6, 150 mM NaCl, 5 mM EDTA, 5 mM β-mercaptoethanol and protease inhibitor cocktail (Sigma). Total proteins were extracted for 40 min at 4°C, then centrifuged for 30 min at 11,000 *g* at 4°C and the supernatant was taken for analysis.

The proteins were precipitated from the crude extract by ammonium sulfate (pH 7.8). Ammonium sulfate was added to samples to a concentration equal to 30% saturation followed by incubation for 12 h at 4°C and centrifugation at 11,000 *g* for 30 min. The precipitate was discarded and ammonium sulfate was added to the supernatant to 70% saturation followed by incubation and centrifugation as indicated above. The obtained precipitate was collected, dissolved in PBS and desalted on a Sephadex G25 column. The obtained preparation was loaded onto an asialofetuin–Sepharose 4B column equilibrated with PBS (pH 7.4). The proteins that bound to the asialofetuin–Sepharose were eluted in 200 mM glycine (pH 3.65) and five fractions were collected. These fractions were analyzed by western blotting.

### Western blot analysis

The proteins were separated by 10–25% gradient SDS-PAGE and transferred to a nitrocellulose membrane (Amersham). Rabbit anti-RTB polyclonal antibodies (diluted 1:1,000) served as the primary antibodies. Anti-rabbit IgG conjugated to alkaline phosphatase was used as the secondary antibody (1:4,000). Blots were treated with nitroblue tetrazolium and 5-bromo-4-chloro-3-indolyl phosphate for visualization.

### Enrichment of crude soluble protein extracts from transgenic plants for immunization assay

The extraction and ammonium sulfate precipitation of TSP from transgenic duckweed lines 81 and G54 were performed as described above for chromatography with immobilized asialofetuin. Precipitated proteins from line 81 were dissolved in PBS buffer and desalted on a Sephadex G25 column equilibrated with 20 mM ammonium bicarbonate buffer (pH 8.2). RTB–M130 protein was quantified in the obtained preparation using asialofetuin-binding ELISA.

The pellets from duckweed line G54 were dissolved in 20 mM Tris–HCl (pH 8.0) and 1 M NaCl, and loaded without desalting onto a Toyopearl butyl chromatography column equilibrated with the same buffer. The proteins were eluted by an inverse linear gradient of NaCl (1.0–0 M) in 20 mM Tris–HCl (pH 8.0). The column was then washed with water, eluting the fusion protein M2e–GUS. M2e–GUS protein was quantified in the obtained preparation by ELISA. The preparations of RTB–M130 and M2e–GUS were then aliquoted and lyophilized and the samples were stored at −70°C until immunization assay.

### Immunization assay

**Figure 2:**
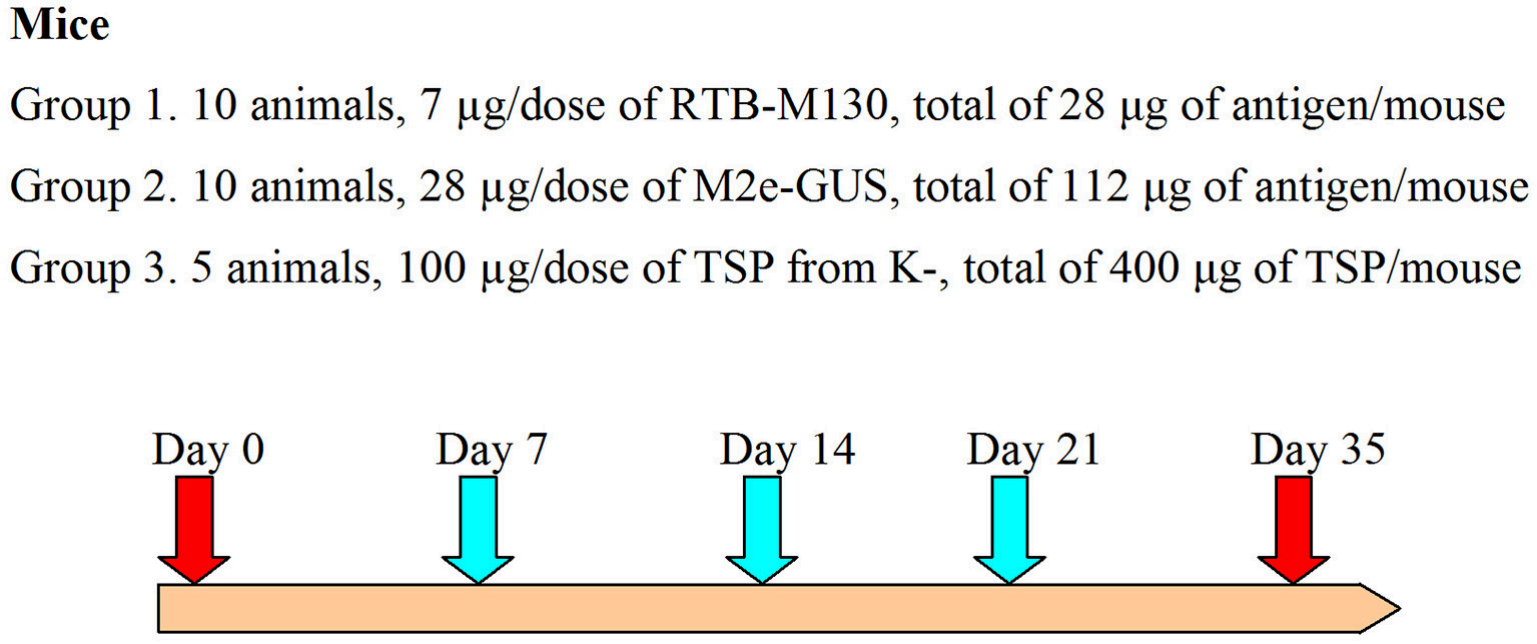
Immunization assay design. The turquoise arrows indicate oral gavage immunizations, red arrows, day 0 and blood samples; K-, denotes non-transformed duckweed plants.

Adult (7- to 9-week-old) male ICR mice weighing 32 ± 6 g were used for the immunization assay. This animal study was conducted in compliance with Good Laboratory Practice (GLP certificate no. G-044), EC Directive 2010/63/EU, and the Russian legislation regulating animal experiments in laboratories for biological testing. Mice were divided into three groups: (1) immunization with RTB–M130 (7 μg/dose, 10 animals); (2) immunization with M2e–GUS (28 μg/dose, 10 animals); and (3) administration of TSP from non-transformed plants (100 μg/dose, 5 animals). Immediately before the assay, immunization preparations were resuspended in sterile PBS and homogenized for 5 min in an ultrasonic bath. The doses (volume 200 μl) were orally administered using a 1-ml syringe equipped with a bulb-tipped gastric gavage needle. The immunizations were carried out on days 0, 7, 14, and 21 (Figure 2). Blood samples were collected 14 days after the fourth immunization, i.e., on day 35, from the mouse's retro-orbital sinus. To evaluate the basal mouse serum response, blood was also collected on day 0, before initiating the oral immunizations.

ELISA was performed to evaluate the immunization response. The microtiter plates were coated with conjugate peptide M2e–keyhole limpet hemocyanin (2 μg/well in PBS) at 4°C for 2 h, then washed and blocked in PBST with 2% (w/v) bovine serum albumin. The mouse sera were initially diluted 32 times in PBS with a subsequent two-fold serial dilution. The plates were incubated with 100 μl/well of mouse serum for 16 h at 4°C. The plates were then washed and incubated with horseradish peroxidase-conjugated anti-mouse IgG (dilution 1:5,000, BioRad) for 2 h at 4°C. The Horseradish Peroxidase Substrate Kit (BioRad) was used, and the reaction was performed as per the manufacturer's instructions. The reaction was stopped after 45 min at room temperature and absorbance was measured at 450 nm. Endpoint antibody titers were defined as the last serum dilution at which the absorption at 450 nm was significantly higher than that of serum of mice immunized with non-transformed plant TSP. Results are expressed as mean optical density (OD) ± *SD* for groups of mice.

### Statistical analysis

The significance of differences in RTB–M130 accumulation between duckweed transgenic lines and in mean antibody levels between groups of immunized mice were analyzed by one-way analysis of variance (ANOVA) test (Statistica 6.1 software; StatSoft Inc).

## Results

### *Agrobacterium*-mediated transformation of duckweed

**Figure 3:**
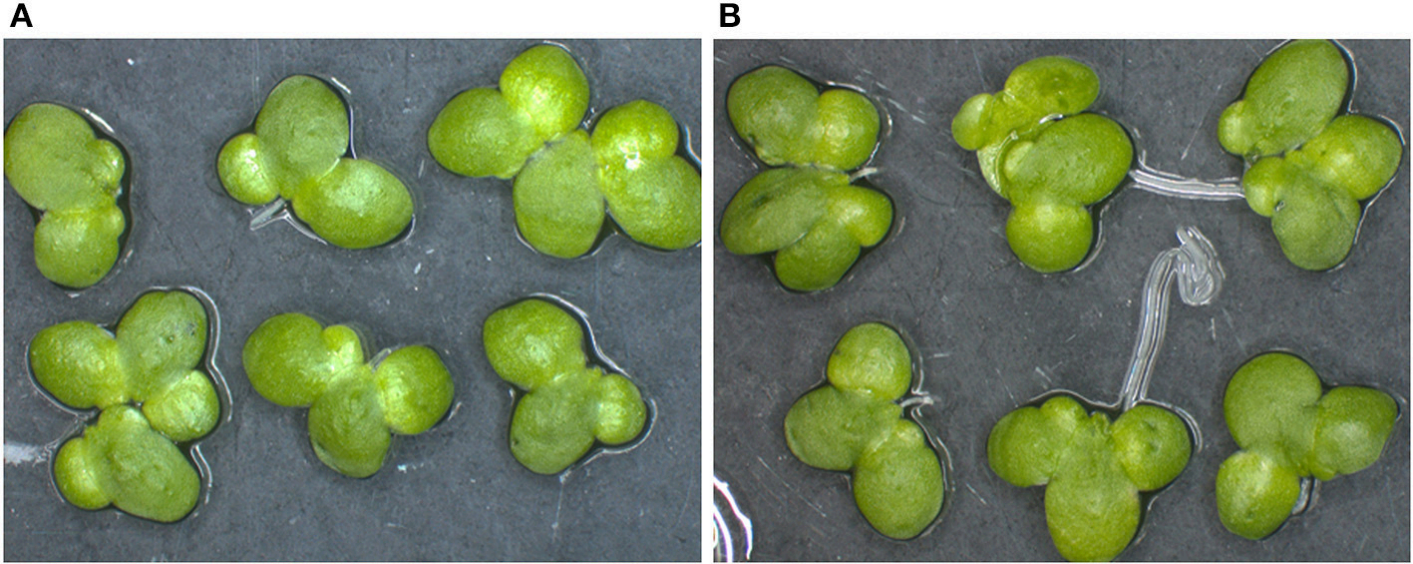
Transgenic and non-transformed duckweed plants. **(A)** Transgenic duckweed plants (line 81) growing in LHFM medium with 10 mg/l kanamycin. **(B)** Non-transformed duckweed plant growing in kanamycin-free medium.

Duckweed plants were transformed with *A. tumefaciens* strain CBE21 carrying the RTB–M130 construct encoding M2e peptide translationally fused to the 3′ end of RTB (Figure 1); 10 g of duckweed callus was used for *Agrobacterium*-mediated transformation. First regenerated fronds, following transformation and regeneration, appeared after 10–12 weeks of cultivation on the medium for transformant selection. Each frond was transferred to a separate culture tube with LHFM medium containing 10 mg/l kanamycin and 200 mg/l cefotaxime for further growth and proliferation. During the cultivation, all plants showing signs of the toxic effect of kanamycin were culled. As a result, 23 independent kanamycin-resistant duckweed lines were obtained. The kanamycin-resistant duckweed plants did not differ morphologically from the non-transformed ones (Figures 3A,B). The development and growth rate of these plants in liquid culture did not differ from the corresponding characteristics of the non-transformed control plants.

### Analysis of RTB–M130 integration and expression in transgenic plants

**Figure 4:**
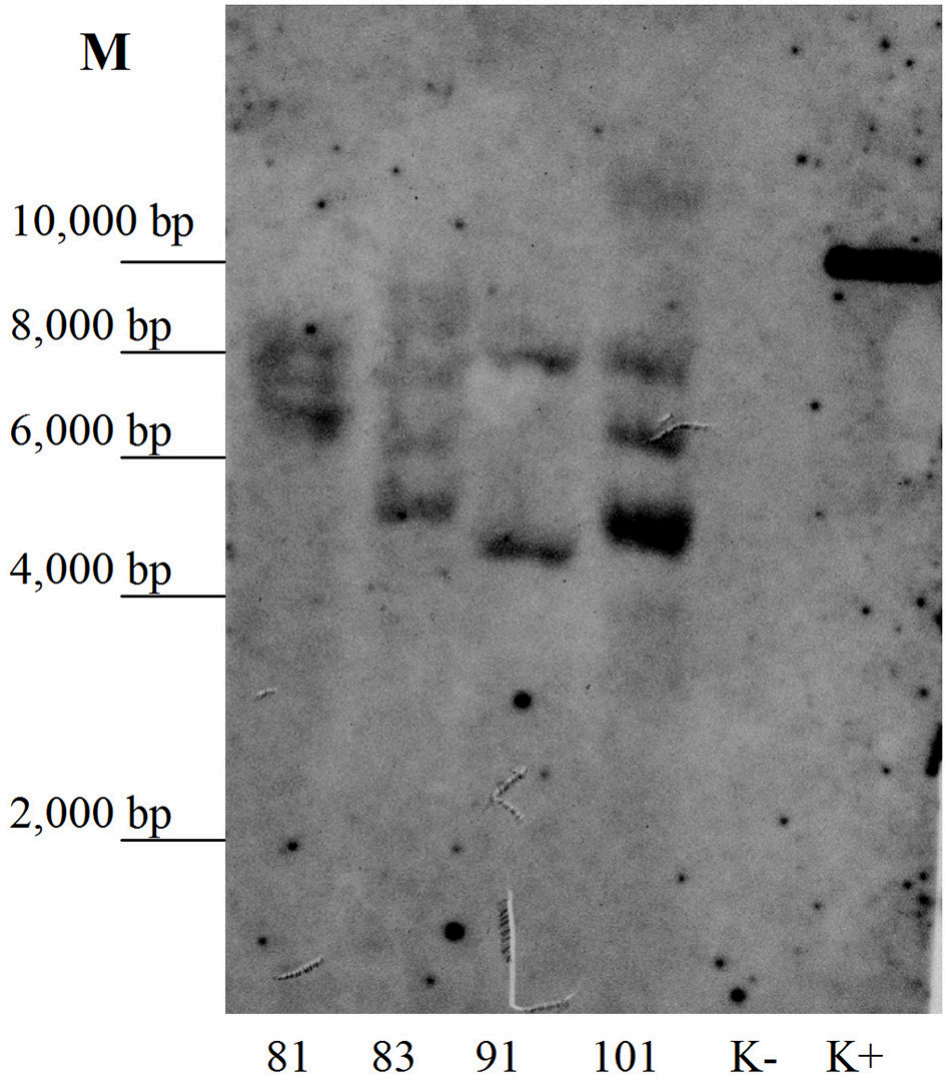
Southern blot analysis of the primary transformants. Numbers denote independent transgenic lines. K–, DNA from non-transformed plants; K+, DNA of plasmid pRTB–M130; M, molecular size markers.

The kanamycin-resistant duckweed lines were analyzed by PCR for the presence of the target RTB–M130 sequence. A DNA fragment of the expected size was amplified from the DNA of all 23 analyzed lines. In DNA samples from non-transformed plants, amplification of the target fragment was not observed. To further confirm the transgenic origin, Southern blot analysis of selected duckweed lines was performed. Genomic DNA was digested with HindIII that cut once within the T-DNA prior to electrophoresis. Results confirmed integration of the nucleotide sequence encoding the RTB–M130 fusion protein into the duckweed genomic DNA (Figure 4). Based on the hybridization profile, there were two (line 91) or three (lines 81, 83, and 101) insertions of the transgene in the studied lines, which is typical for *Agrobacterium*-mediated transformation. The DNA from non-transformed plants failed to hybridize to the probe.

**Figure 5:**
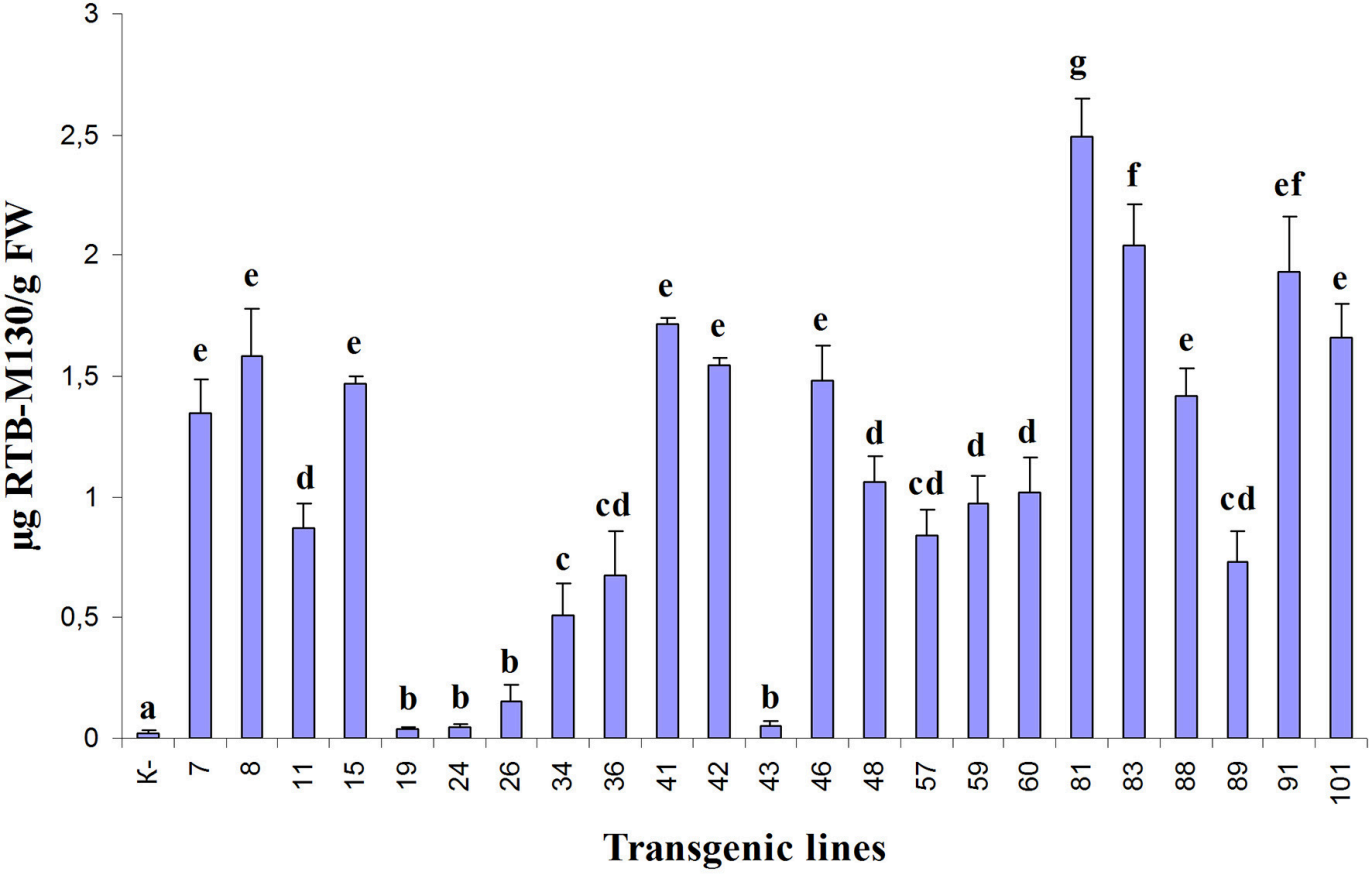
Quantification of RTB–M130 fusion protein in transgenic duckweed plants by asialofetuin-binding ELISA using anti-RTB antibody. K–, non-transformed plants. Numbers denote transgenic lines. Error bars indicate ± *SD*. Different letters denote significant differences for mean amount of RTB–M130 among duckweed transgenic lines (*P* < 0.05). Quantification of RTB–M130 was performed in three biological replicates.

Asialofetuin-binding ELISA revealed the presence of ricin B subunit in all analyzed transgenic duckweed lines (Figure 5). In three transgenic lines (19, 24, and 43), only negligible amounts of RTB were detected. The accumulation of the fusion protein RTB–M130 in transgenic lines varied from 0.25 μg/g (line 26) to 2.5 μg/g FW (line 81), corresponding to 0.0006 and 0.01% of TSP, respectively. A relatively high level of RTB–M130 expression was observed in lines 81, 83, 91, and 101—about 2.0 μg/g FW (0.01% of TSP). Based on the obtained data, we selected transgenic lines with high and average levels of accumulated product for further studies (lines 41, 60, 81, 83, 91, and 101).

**Figure 6:**
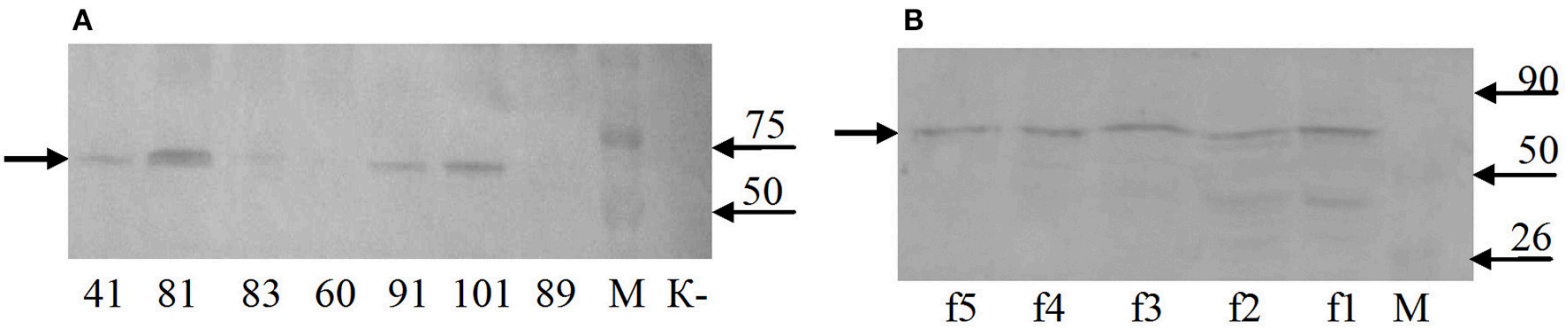
RTB–M130 protein expression in transgenic duckweed lines. **(A)** Western blot analysis of protein samples from plants. **(B)** Western blot analysis of fractions after chromatography with immobilized asialofetuin (transgenic line 81). K–, non-transformed duckweed plants; M, molecular size markers. Numbers denote transgenic lines; f1–f5, chromatography fractions (transgenic line 81). Arrow on the left indicates immunoreactive band with molecular mass of ~70 kDa.

Western blot analysis of total protein samples from the selected transgenic duckweed lines revealed the presence of an immunoreactive band of ~70 kDa (Figure 6A). The 32-kDa bands corresponding to the fusion protein RTB–M130 were not observed. Immunoreactive bands of these weights were not detected in the protein samples from non-transformed control plants. An additional experiment was performed to evaluate the interaction between RTB–M130 and asialofetuin by affinity chromatography (Figure 6B). Western blot analysis of protein fractions from transgenic line 81, obtained following affinity chromatography with immobilized asialofetuin, using antibodies to RTB, revealed the presence of an immunoreactive band at around 70 kDa. The corresponding bands were not detected in protein fractions from non-transformed plants. From these results, we hypothesized that RTB–M130 is expressed as an aggregate with a molecular mass of about 70 kDa. Recombinant RTB–M130's ability to bind to the glycoprotein asialofetuin suggested its ability to bind to cellular receptors.

### Mouse-immunization assay

In a preliminary experiment, laboratory mice were reluctant to eat duckweed—fresh or lyophilized, alone or mixed with dry feed. This made it impossible to immunize the mice by feeding duckweed biomass. To overcome this, immunization with partially purified preparations of TSP from transgenic duckweed was performed by gastric gavage.

**Figure 7:**
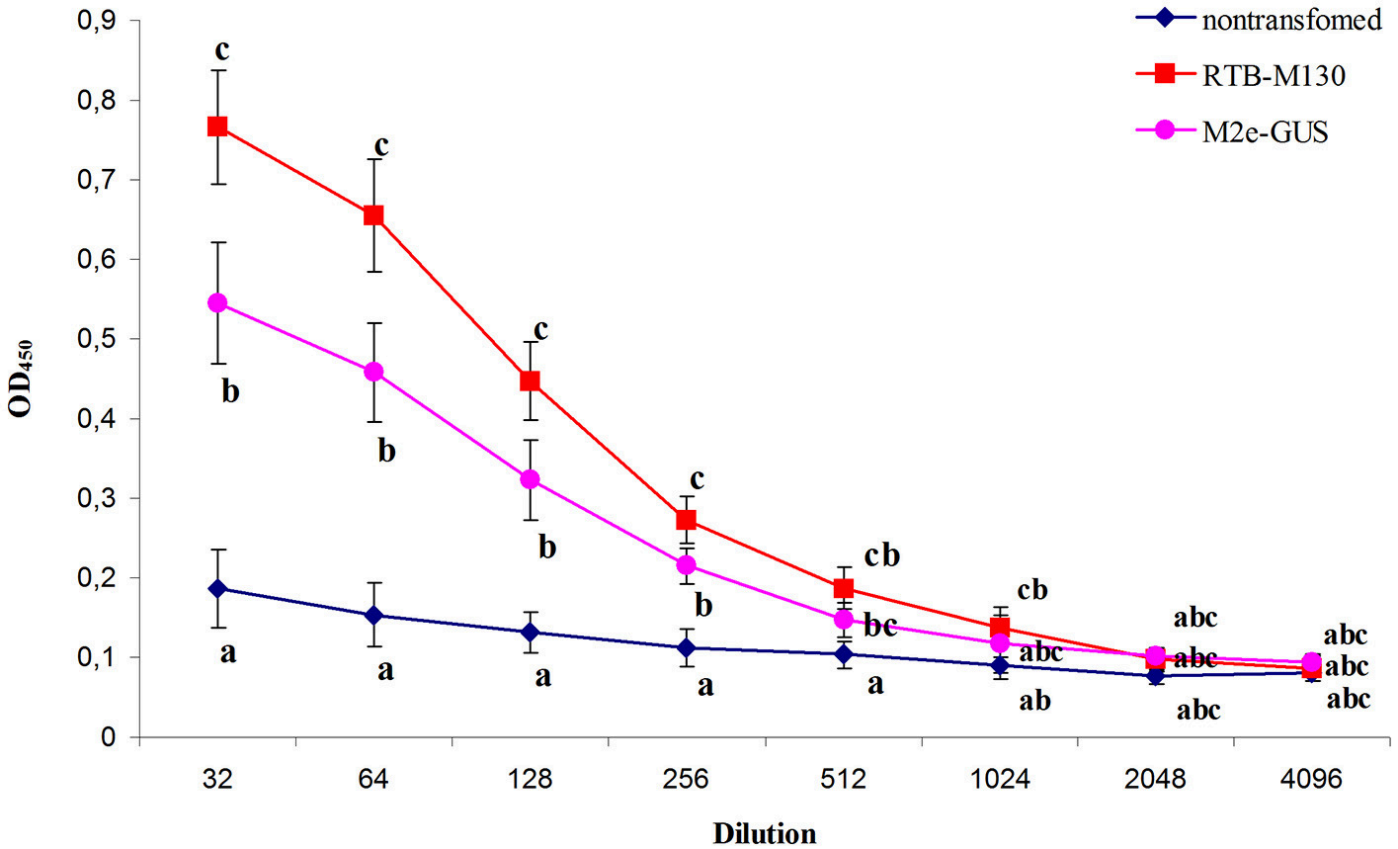
Immune response of mice after oral immunization with total soluble protein from RTB–M130- and M2e–GUS-transgenic duckweed. Total protein from non-transgenic plants was used as a control in the immunization experiments. Levels of IgG against M2e were evaluated by ELISA. Results are presented as mean OD values ± *SD*. Different letters indicate significant differences between immunized groups (*P* < 0.05).

As expected, in mice immunized with TSP from non-transgenic plants, no antibody to M2e was detected. Moreover, no animals had detectable levels of M2e-specific IgG in their preimmune serum, indicating that there had been no prior exposure to the influenza virus. Plant-derived RTB–M130 and M2e–GUS fusion proteins elicited a specific immune response (Figure 7). IgG against peptide M2e accumulated in all mice immunized with RTB–M130 and M2e–GUS, indicating that the fusion proteins are internalized through the mucosal lining and able to induce the immune system.

As shown in Figure 7, mice gavaged with RTB–M130 developed specific anti-M2e IgGs with an endpoint ELISA titer of 1024. Immunization of animals with M2e–GUS also elicited an immune response, with an anti-M2e IgG titer of 512. The immune response in this group was markedly weaker, even though each mouse received 112 μg M2e–GUS in its immunization cycle, compared to 28 μg/mouse of RTB–M130. The obtained results confirmed retention of the plant-derived RTB's adjuvant properties, and weak immunogenicity of peptide M2e with GUS as the adjuvant. In summary, these results confirm the possibility of expressing M2e peptide in nuclear-transformed duckweed and reveal a much higher level of anti-M2e IgG in orally immunized mice when M2e is fused to RTB as compared to GUS.

## Discussion

We obtained 23 different lines of duckweed with confirmed transgenic status; the fusion protein RTB–M130 was detected in all of these lines. Transgenic duckweed plants did not differ morphologically from their non-transformed counterparts. Expression of the fusion protein had no effect on growth rate in liquid culture or on plant TSP content. Accumulation of RTB–M130 according to asialofetuin-binding ELISA ranged from 0.25 to 2.5 μg/g FW of duckweed (0.0006–0.01% of TSP). Strong variations in recombinant protein expression in independently derived transgenic lines are common (Richter et al., 2000; Sharma and Sharma, 2009). They are often related to differences in the number of transgene copies or in the genome position into which the foreign DNA has integrated. Both of these factors are relevant to the transgenic duckweed lines that we obtained.

A few studies have been performed on the expression of RTB in heterologous systems, alone or as part of a fusion protein. According to Reed et al. (2005), the accumulation of ricin B subunit in transgenic tobacco plants averaged 0.007% of TSP (about 0.35 μg RTB functional equivalent per 1 g leaf FW). In Woffenden's, Ñopo, Cramer, Dolan and Medina-Bolivar (2008), tobacco plants were transformed with the nucleotide sequence encoding RTB in translational fusion with synthetic genes F1 and V of *Yersinia pestis*. The fusion protein RTB–F1–V accumulated in the leaves of transgenic plants at a level of about 45 ng RTB functional equivalent per 1 g FW (corresponding to 0.0015–0.0025% of TSP). Somewhat higher levels of RTB expression were observed by Choi et al. (2006)—up to 0.03% of TSP. In that study, ricin B subunit was fused to the C-terminal end of capsid glycoprotein VP7 of monkey rotavirus SA11, and fusion protein VP7–RTB was expressed in potato tubers. Transformation of tobacco with cDNA encoding the full-length preproricin resulted in its accumulation in leaf tissues to 0.25% of TSP (Sehnke et al., 1994). In those studies, asialofetuin-binding ELISA showed that the ricin B subunit was functionally active, indicating its correct processing and glycosylation in the heterologous systems. Interestingly, in Singh et al. (2015), the accumulation of fusion protein rabies glycoprotein (RGP)–RTB in tomato hairy roots reached 1.14% of TSP. RGP–RTB was expressed as a monomer; its functional viability was confirmed by asialofetuin-binding ELISA.

It should be noted that the accumulation of GUS in line G54 was almost a thousand times greater than that of RTB–M130 in our transgenic lines. A high level of GUS accumulation is fairly common. For example, in a study by Butaye et al. (2004), GUS accumulation in the cytoplasm reached 10% of TSP. It seems that there, the high expression of GUS was due to its high stability in the cytoplasm (half-life in living mesophyll protoplasts of ~50 h; Jefferson et al., 1987), allowing it to accumulate in large quantities.

A low level of accumulation seems to be characteristic of RTB expressed in nuclear-transformed heterologous systems. This is most likely due to its rapid degradation in the secretory pathway of plant cells. In experiments with tobacco (both protoplasts and nuclear-transformed plants), the bulk of the newly synthesized RTB disappeared in the early secretory pathway, presumably as a result of proteolysis in the endoplasmic reticulum. The recombinant RTB degraded very rapidly, and its accumulation in the protoplasts was therefore very low (Chamberlain et al., 2008). The plant cell proteolytic system likely recognizes the recombinant RTB as a misfolded or unassembled polypeptide that is then targeted for elimination. Kiani et al. (2013) observed a high level of RTB expression in transgenic cotton plants. In those experiments, RTB was fused to the C terminus of the Cry1Ac protein (a δ-endotoxin of *Bacillus thuringiensis*). The fusion protein Cry1Ac–RTB was targeted to chloroplasts by a transit peptide from petunia, and its accumulation in the plants was estimated at 3.5% of TSP. The high level of accumulation of Cry1Ac–RTB might have been because it was transported into chloroplasts without entering the endoplasmic reticulum, thereby avoiding degradation.

In our experiments, fusion protein RTB–M130 was detected as a band with a molecular mass of about 70 kDa (calculated mass of the protein without the N-terminal signal peptide–32.6 kDa), and it was therefore presumably in aggregated form. Similar expression behavior of RTB in a fusion protein was observed by Carter et al. (2010), where it was fused with the C-terminal end of proinsulin (Ins) in transgenic potato tubers. Those authors showed that the fusion protein Ins–RTB apparently aggregates in large molecular complexes, being detected as bands of more than 200 kDa (estimated mass of Ins–RTB-−38.2 kDa). Attempts to dissociate these aggregates into monomers were unsuccessful. The authors believed that aggregation of Ins–RTB occurs due to the formation of a large number of chaotic intermolecular disulfide bonds. Nevertheless, Ins–RTB retained the ability to bind to asialofetuin (Carter et al., 2010). In our experiments, RTB–M130 also retained the ability to bind to asialofetuin and elicit an immune response in mice. Thus, aggregation of RTB does not influence its ability to bind to its corresponding cell receptors and act as an adjuvant.

Aggregation has only been observed when RTB is fused with small proteins—M130 peptide (3.3 kDa) or proinsulin (9.4 kDa). In experiments where RTB is fused with a relatively large protein, e.g., VP7 (34 kDa; Choi et al., 2006), F1 and V proteins (15 and 37 kDa, respectively; Woffenden et al., 2008), green fluorescent protein (26 kDa; Medina-Bolivar et al., 2003), RGP (56.5 kDa; Singh et al., 2015), Cry1Ac protein (65 kDa; Kiani et al., 2013), or expressed as ricin alone (Sehnke et al., 1994), there is no aggregation, and fusion proteins have the expected molecular mass. Size and structure optimization of the RTB partner in fusion proteins may prevent or substantially reduce its aggregation when expressed in heterologous systems.

As already noted, the M2e peptide is a poor immunogen, and requires an adjuvant to become sufficiently immunogenic (Rossman and Lamb, 2011). In most cases, the M2e peptide is expressed in heterologous systems in fusion with some adjuvant protein. To obtain a mucosal immune response, such adjuvants have included CTA1–DD (subunit A of cholera toxin fused to the D domain of protein A from *Staphylococcus aureus* (De Filette et al., 2005, 2006), subunit B of cholera toxin (Shim et al., 2011), hepatitis B virus core antigen (Ravin et al., 2012), and C-terminal domain of heat-shock protein 70 from *M. tuberculosis* (Ebrahimi and Tebianian, 2011).

The immunogenicity of the RTB–M130 fusion protein and the adjuvant effect of RTB were confirmed in our study. Immunization of mice with RTB–M130 elicited a detectable immune response. Immunization with M2e–GUS elicited a weaker immune response, even though the antigen dose was significantly higher than when immunized with RTB–M130. When the mice were immunized with RTB–M130, the anti-M2e antibody titer was clearly insufficient for complete protection against the influenza virus. We suggest increasing the immunization dose of RTB–M130. To this end, it is necessary to increase the level of RTB–M130 accumulation in transgenic plants. We believe that this can be achieved by (1) optimizing the size and structure of the antigenic part in the fusion protein and (2) selecting the optimal compartment for its accumulation.

Our experiments demonstrated the feasibility of expressing M2e peptide fused to ricin B subunit in nuclear-transformed duckweed plants, with no impact on plant morphology or growth rate. Oral immunization with the recombinant RTB–M130 protein elicited an immune response and induced anti-M2e antibodies in mice. The immunization assay also confirmed that the ricin B subunit retains its adjuvant properties in the fusion protein, enhancing induction of anti-M2e antibodies in the vaccinated animals. The present study demonstrates that transgenic duckweed plants can produce quality antigen toward the development of an edible “universal” vaccine against influenza viruses.

## Author contributions

TM and IT: Performed genetic transformation and *in vitro* duckweed culture; LS, OK, and LV: Analyzed target protein expression, including western blotting and chromatography, and prepared the samples for mouse immunizations; AF and IT: Performed the PCR and Southern blot analyses; AM and ER: Performed the immunization assays; SD and AV: Planned the experiments, provided general guidance, and prepared the article.

### Conflict of interest statement

The authors declare that the research was conducted in the absence of any commercial or financial relationships that could be construed as a potential conflict of interest. The reviewer, RV, and handling Editor declared their shared affiliation.
