# Supplementary material for: Expression and Immunogenicity of M2e Peptide of Avian Influenza Virus H5N1 Fused to Ricin Toxin B Chain Produced in Duckweed Plants
Source: Front Chem. 2018 Feb 13;6:22. doi: 10.3389/fchem.2018.00022 (PMC5816751; doi:10.3389/fchem.2018.00022)
Supplement: Supplementary file 1 [file Table1.doc]

**Supporting table**

| Primer | Amplified nucleotide sequence | Nucleotide sequence, 5’→3’ | PCR regime |
| --- | --- | --- | --- |
| RTB/F | ricin B subunit | cg**tctaga**accggtgctgatgtttgtatggatcctgag - F | 1 min at 94°C, 30 s at 63°C, 2 min at 72°C; 30 cycles |
| RTB/R |  | cgt**tctaga**accggtctgcaagagagtaatctgtctatca - R |  |
| M130f | peptide M130 | att**tctaga**atgtccctcctcactgaag - F | 1 min at 94°C, 30 s at 62°C, 30 s at 72°C; 30 cycles |
| M130r |  | g***gagctc***cgccgccaccaccaaggggt - R |  |
| RBspF | signal peptide of PR1a protein | agaaccggtatgggatttgttctcttttc - F | 1 min at 94°C, 30 s at 57°C, 30 s at 72°C; 30 cycles |
| RBspR |  | agcaccggtagaattttgggcacgg - R |  |
| 5727 | CaMV 35S promoter - peptide M130 | aagggatgacgcacaatc - F | 1 min at 94°C, 30 s at 56°C, 1 min at 72°C, 30 cycles |
| М130r |  | gggatcccgccgccaccaccaaggggt - R |  |
| virC1 | gene virC *A. tumefaciens* | gcactatctacctaccgctacgtcatc - F | 1 min at 94°C, 30 s at 59°C, 1 min at 72°C, 30 cycles |
| virC2 |  | gttgtcgatcgggactgtaaatgtg - R |  |

**S1 Table. PCR Regimes and Nucleotide Sequences of Used Primers**.

The cloning site *XbaI* is in bold, *SacI* in bold italics, site *AsiAI* is underlined. F –forward primer, R – reverse primer.
